# Soil bacterial community composition altered by increased nutrient availability in Arctic tundra soils

**DOI:** 10.3389/fmicb.2014.00516

**Published:** 2014-10-02

**Authors:** Akihiro Koyama, Matthew D. Wallenstein, Rodney T. Simpson, John C. Moore

**Affiliations:** ^1^Natural Resource Ecology Laboratory, Colorado State UniversityFort Collins, CO, USA; ^2^Department of Biology, Colorado State UniversityFort Collins, CO, USA; ^3^Department of Ecosystem Science and Sustainability, Colorado State UniversityFort Collins, CO, USA

**Keywords:** soil, bacteria, fungi, Arctic tundra, fertilization, nitrogen, phosphorus, mycorrhizae

## Abstract

The pool of soil organic carbon (SOC) in the Arctic is disproportionally large compared to those in other biomes. This large quantity of SOC accumulated over millennia due to slow rates of decomposition relative to net primary productivity. Decomposition is constrained by low temperatures and nutrient concentrations, which limit soil microbial activity. We investigated how nutrients limit bacterial and fungal biomass and community composition in organic and mineral soils within moist acidic tussock tundra ecosystems. We sampled two experimental arrays of moist acidic tussock tundra that included fertilized and non-fertilized control plots. One array included plots that had been fertilized annually since 1989 and the other since 2006. Fertilization significantly altered overall bacterial community composition and reduced evenness, to a greater degree in organic than mineral soils, and in the 1989 compared to the 2006 site. The relative abundance of copiotrophic α-Proteobacteria and β-Proteobacteria was higher in fertilized than control soils, and oligotrophic Acidobacteria were less abundant in fertilized than control soils at the 1989 site. Fungal community composition was less sensitive to increased nutrient availability, and fungal responses to fertilization were not consistent between soil horizons and sites. We detected two ectomycorrhizal genera, *Russula* and *Cortinarius* spp., associated with shrubs. Their relative abundance was not affected by fertilization despite increased dominance of their host plants in the fertilized plots. Our results indicate that fertilization, which has been commonly used to simulate warming in Arctic tundra, has limited applicability for investigating fungal dynamics under warming.

## INTRODUCTION

Arctic tundra soils represent one of the largest terrestrial carbon (C) pools on earth (Loya and Grogan, 2004; Tarnocai et al., 2009), due to low rates of organic matter decomposition relative to net primary productivity over millennia (Marion and Oechel, 1993). Decomposition of soil organic matter (SOM) in the Arctic is constrained by low temperature, anoxic conditions due to poor drainage associated with underlying permafrost, and nutrient limitations to microbial heterotrophic activity.

Arctic tundra may be among the most nitrogen (N) limited ecosystems in the world, and N often limits both plant productivity and microbially driven SOM decomposition. Much of our understanding of the role of N in Arctic ecosystems comes from long-term field N-addition experiments. Long-term experimental N-addition has led to a significant increase in plant biomass in different types of tundra ecosystems including moist acidic tundra (Chapin and Shaver, 1985; Chapin et al., 1995; Shaver et al., 2001), wet sedge (Shaver et al., 1998), and dry heath (Gough et al., 2002). Nutrient addition has also accelerated SOM decomposition (Mack et al., 2004; Nowinski et al., 2008). Specifically, in moist acidic tundra, where above-ground net primary productivity is strongly limited by N (Shaver and Chapin, 1986; Chapin et al., 1995), 18 years of chronic N and phosphorus (P) fertilization accelerated SOM decomposition, resulting in net C loss, despite significantly increased plant biomass, relative to non-fertilized control (Mack et al., 2004). At an adjacent study site with a similar design and treatment regime, Hobbie and Gough (2004) demonstrated that long-term N and P fertilization in moist acidic tundra accelerated litter decomposition. This finding was supported by Koyama et al. (2013) who found that the fertilization stimulated soil microbial production of C-degrading enzymes, which are proximate drivers of soil organic carbon (SOC) decomposition in the same study site for Hobbie and Gough (2004). Thus, N availability plays a key role in determining the balance between net primary productivity and SOM decomposition, and whether tundra ecosystems are a net C sink or source.

Soil microbial communities in Arctic tundra, which are responsible for SOM decomposition, are as diverse as those found in other biomes (Neufeld and Mohn, 2005; Chu et al., 2010), despite the harsh environmental conditions. Soil microbial diversity in Arctic tundra is likely to be driven by many environmental factors, including N availability. In Arctic tundra soils, where N is stored in numerous chemical forms of different recalcitrance (Schulten and Schnitzer, 1997), the diversity may be an important aspect in N cycling as the diversity can increase nutrient recycling efficiency via greater intensity in organic compound exploitation and/or complementary functional niches (Loreau, 2001).

To what extent does N-availability structure soil microbial communities and influence microbially mediated functions in an Arctic tundra ecosystem? To address this question, we considered how N availability directly and indirectly influenced the microbial community structure (abundance and diversity). Increased levels of readily available N (e.g., NH_4_^+^, NO_3_^-^, urea) via fertilization can favor nitrophilic over nitrophobic taxa, resulting in a less even community composition. For instance, Lilleskov et al. (2001) assessed nitrophobic and nitrophilic ectomycorrhizal taxa using a N deposition gradient in forests dominated by *Picea glauca* (Moench) Voss in Alaska, USA. The study showed that some genera, including *Cortinarius, Russula, Tricholoma, Lactarius,* and *Hebeloma*, were categorized as nitrophobic for being less abundant in forests with high N deposition. Other taxa, including *Lactarius theiogalus, Laccaria, Paxillus involutus,* and *Hygrophorus olivaceoalbus*, were categorized as nitrophilic as their abundance did not decline in response to the N deposition (Lilleskov et al., 2001). Increased N availability can stimulate microbial production of C-degrading extracellular enzymes (Koyama et al., 2013), resulting in increased available C, which in turn can restructure heterotrophic microbial communities.

Nitrogen can also indirectly affect microbial communities via increased net primary productivity of vegetation, especially shrubs (Chapin and Shaver, 1985; Chapin et al., 1995; Shaver et al., 2001). In moist acidic tussock tundra ecosystems, N and P addition significantly increased cover of deciduous shrubs, primarily *Betula nana*, in only 2–3 years (Shaver et al., 2001; Hobbie et al., 2005). Hobbie et al. (2005) reported that mean coverages of deciduous shrubs 2–6 years after the initiation of annual fertilization treatments were 71 and 26% for fertilized and control plots, respectively. This greater coverage of deciduous shrubs with fertilization led to significantly greater above-ground biomass and almost doubled above-ground net primary productivity of vascular plants compared to control (Hobbie et al., 2005; Gough et al., 2012). Shaver et al. (2001) reported that a 15-year fertilization of N and P increased above-ground biomass and net primary productivity of vascular plants by 2.5 times. Increased nutrient availability also affected root structures in moist acidic tussock tundra. Sullivan et al. (2007) reported that long-term fertilization over a decade significantly increased fine root biomass, but decreased production of fine roots at the community level. This result was attributed to the replacement of annual fine root system of tussock, *Eriophorum vaginatum*, with longer-lived fine roots of *B. nana* (Sullivan et al., 2007). Changes in vegetation of this sort can alter the quantity and quality of plant materials affecting the abundance and activities of certain microbial taxa. Increased net primary productivity can result in increased labile C flow to soils via root exudates (Coleman, 1985; Wall and Moore, 1999; Moore et al., 2003). Increased availability of labile C has been shown to favor bacteria and their consumers over fungi and their consumers (Moore et al., 2003), and at a finer resolution, favor copiotrophic over oligotrophic microbes (Fierer et al., 2007). The increased C flow to soils via plant roots has also been shown to stimulate biological activities in deeper mineral soils (Sistla et al., 2013).

Changes in the types of roots and root biomass affect mycorrhizal fungi associations as well (Hobbie, 2006; Hobbie and Hobbie, 2006; Hobbie et al., 2009). In Arctic tundra ecosystems, however, responses of root biomass to fertilization have varied from no change (Mack et al., 2004; Gough et al., 2012) to significant increase (Jonasson et al., 1999; Nadelhoffer et al., 2002) at a community level. Clemmensen et al. (2006) found that a 13-year fertilization of N and P significantly increased fine root biomass of shrubs associated with ectomycorrhizal fungi in moist acidic tundra, but no such increase occurred in heath tundra. Such a range in responses of root biomass to added N raises uncertainty in predicting effects on the mycorrhizal community. The relative abundance of mycorrhizal fungi can increase along with increased root biomass (Clemmensen et al., 2006), but at some point mycorrhizal infection rates decrease with fertilization (Treseder, 2004; Johnson et al., 2006; Hoeksema et al., 2010).

Addition of nutrients, typically N and P, has been used to surrogate a warming effect in Arctic tundra ecosystems (Mack et al., 2004; Aerts, 2010). The rationale is that warming often increases nutrient availability by accelerating decomposition of SOM (Chapin et al., 1995; Hartley et al., 1999; Rustad et al., 2001; Schmidt et al., 2002; Schimel et al., 2004; Aerts et al., 2006). However, Rinnan et al. (2007, 2013) found that long-term warming and fertilization treatments over a decade affected soil microbial communities differently in a Swedish Arctic tundra ecosystem, concluding that nutrient addition might not be a suitable means to mimic warming to assess soil microbial dynamics.

In this study, we investigated how bacterial and fungal biomass and their community composition were altered by long-term fertilization treatments in a moist acidic tussock tundra ecosystem of the Alaskan Arctic. We selected two study sites with different fertilization durations; one site had been fertilized for 23 years as of the sample collection since 1989 (1989 site) and the other for 6 years since 2006 (2006 site). At the two sites, we collected soil samples from two levels of treatments, fertilized (10 *g* N⋅m^-2^⋅year^-1^ and 5 *g* P⋅m^-2^⋅year^-1^) and control. We predicted that fertilization had altered bacterial and fungal community composition and decreased diversity through its influence on plant community structure and the quality and quantity of plant by-products (e.g., roots, detritus, rhizodeposition including root exudates), and that these effects were greater in the 1989 than 2006 sites, and in organic compared to mineral soils. We also predicted that the fertilization treatments had significantly increased the relative abundance of copiotrophs (e.g., α-Proteobacteria) and reduced the abundance of oligotrophs (e.g., Acidobacteria) through increased nutrient availability as a direct fertilization effect and/or increased C input via stimulated net primary productivity as an indirect fertilization effect (Ramirez et al., 2010). Finally, we predicted that the fertilization treatments altered fungal community composition by increasing the relative abundance of mycorrhizal fungi associated with shrubs, including *B. nana*, which became dominant over other vegetation forms in response to fertilization (Shaver et al., 2001; Hobbie et al., 2005). Our results contribute to the debate as to whether nutrient addition is a suitable means to mimic a warming effect in Arctic tundra ecosystems (Mack et al., 2004; Rinnan et al., 2007, 2013; Aerts, 2010).

## MATERIALS AND METHODS

### STUDY SITE AND SAMPLE COLLECTION

Soils were collected from the Arctic Long-Term Ecological Research (LTER) site at Toolik Lake, AK, USA (68°38’N, 149°38’W) in late July 2011. The soils in moist acidic tussock tundra on the hillslopes near Toolik Lake are classified as Typic Aquiturbels (Bockheim, 2007), consisting of an organic horizon of varying thickness overlaying a mineral soil with imbedded permafrost. The average annual temperature and precipitation are –7°C and 400 mm, respectively, with approximately half of the annual precipitation as snow. The growing season is limited to between 50 and 70 days in July and August when a mean temperature is ∼10°C. Moist acidic tussock tundra is the dominant habitat type where vegetation consists of graminoids (*E. vaginatum* and *Carex microchaeta*), deciduous shrubs (*B. nana*), evergreen shrubs (*Ledum palustre* and *Vaccinium vitis-idaea*), and mosses (*Sphagnum spp.*, *Hylocomium splendens*, and *Aulacomnium spp.*; Shaver and Chapin, 1991; Chapin et al., 1995; McKane et al., 1997; Gough et al., 2007). *B. nana* is a shrub species with obligate symbiosis with ectomycorrhizal fungi (Molina et al., 1992) including *Russula* and *Cortinarius* spp. Evergreen shrubs, *Ledum palustre* and *V. vitis-idaea* are ericaceous associated with ericoid mycorrhizal fungi dominated by members of the order *Helotiales* (Walker et al., 2011).

Samples were collected from two experimental sites established in 1989 and 2006 that contained annual fertilization treatments and controls. The two different sites were located on adjacent hillslopes in moist acidic tussock tundra, 175 m apart from each other. The 1989 site is arranged in a randomized complete block design with four blocks, each containing a fertilization treatment (10 *g* N.m^-2^.year^-1^ as NH_4_NO_3_ and 5 *g* P.m^-2^.year^-1^ as P_2_O_5_) and control. The 2006 site is also arranged in a randomized complete block design with three blocks, each containing a control and a fertilization treatment applied at the same rate as the 1989 site. Responses of above-ground vegetation to the fertilization treatments at the sites assessed in late July and early August of 2011 were similar to those in the same area reported in previous studies (Chapin and Shaver, 1985; Chapin et al., 1995; Shaver et al., 2001); deciduous shrubs became dominant over other functional types with fertilization relative to non-fertilized controls in the 1989 site, and such vegetation shift was in transition in the 2006 sites (unpublished data). Two sub-samples were collected from each plot. Each soil sample was separated into three horizons: organic, organic/mineral interface and mineral soils based on organic matter content and degree of decomposition. Depths of the organic soils varied from 6 to 12 cm, and the interface from 4 to 15 cm. Mineral soils were collected from the top 5 cm of the horizon beneath the interface soils. Samples were frozen at –20°C at Toolik Field Station up to 2 weeks, transported in a cooler on dry ice to the EcoCore Analytical Laboratory at the Natural Resource Ecology Laboratory, Colorado State University, Fort Collins, CO, USA, and stored at -80°C until DNA was extracted from the soils.

### SOIL PROPERTY MEASUREMENTS

Soil samples were quantified for soil properties, including bulk density, soil water content, SOC, and total N contents. All the soil properties, except bulk density, were previously reported by Koyama et al. (2013) and summarized in **Table 1**. Soil water content was determined by drying soil samples at 105°C for 48 h. To measure SOC and total N contents, samples were first dried out at 60°C, and ground finely using a Brinkmann Retsch mill (Haan, Germany). Total C and N contents of the ground samples were quantified via dry combustion using a LECO TruSpec®; (Leco Corporation, St. Joseph, MI, USA). To measure bulk density, we used sub-samples of known dimensions (i.e., width × depth × height in cm) sliced from harvested organic and mineral soils. The sub-samples were dried at 60°C for up to 10 days until their weights did not decline further. Bulk density of each sample was calculated using its volume (cm^3^) measured upon harvest, and its dry weight (*g*).

**Table 1:** Soil properties (mean ± 1 se) assessed for microbial community compositions in this study.

Site	1989 Site		2006 Site	
Treatment	Control	Fertilized	Control	Fertilized
**Organic**
Bulk density (*g* cm^-3^)	0.13 ± 0.03	0.31 ± 0.22	0.07 ± 0.01	0.11 ± 0.03
Horizon depth (cm)	11.88 ± 3.63	9.50 ± 1.55	4.67 ± 0.44	4.50 ± 1.04
SOC (%)	39.34 ± 1.98	39.18 ± 5.09	45.74 ± 0.81	33.90 ± 6.50
Total *N* (%)	0.85 ± 0.11	1.81 ± 0.24	0.89 ± 0.08	1.24 ± 0.26
C:N ratio	48.45 ± 5.70	21.69 ± 0.82	52.10 ± 4.98	27.51 ± 2.37
**Mineral**
Bulk density (*g* cm^-3^)	1.09 ± 0.22	0.97 ± 0.36	0.95 ± 0.14	1.08 ± 0.26
Horizon depth (cm)	10.75 ± 3.25	15.25 ± 2.95	16.67 ± 3.33	17.67 ± 2.33
SOC (%)	8.75 ± 2.42	9.05 ± 3.08	3.14 ± 0.51	3.70 ± 0.79
Total *N* (%)	0.39 ± 0.11	0.44 ± 0.15	0.17 ± 0.03	0.20 ± 0.03
C:N ratio	22.52 ± 1.34	20.80 ± 0.33	18.93 ± 0.62	18.35 ± 0.72

### MICROBIAL BIOMASS

Bacterial and fungal biomass was estimated using a direct count method modified from Bloem (1995) and Frey et al. (1999). A five-gram soil sample was added to 45 mL filtered and sterilized (autoclaved) de-ionized water and blended in a Waring blender for 1 min. A 1 mL aliquot was immediately added to 9 mL of filtered sterile de-ionized water, from which five 10 μL sub-samples were pipetted onto one side of a sterile, 10-well (6 mm) microscope slide and allowed to air dry. Separate slides were used for bacteria and fungi, but the 10 μL sub-samples on each slide were from the same soil solution. Bacterial samples were then stained with DTAF (5-(4,6 dichlorotriazin-2-yl) aminofluorescein) while fungal samples were stained with calcifluor M2R fluorescence brightener (Bloem, 1995), rinsed and allowed to air dry. A drop of immersion oil (type FF) was placed on each well and a cover slip was affixed to each slide. All finished samples were stored in the dark at 4°C until direct counts could be made. Bacterial cell counts and fungal hyphal length estimation were made using a confocal microscope at 1500 and 400× magnification, respectively. Bacterial cell counts were converted to bacterial biomass assuming an average dry weight of 6.65 × 10^-13^ *g* C per bacterial cell (Ilic et al., 2001). Fungal hyphal lengths were estimated using a grid intercept technique by counting the number of times hyphae crossed an ocular lens grid. Fungal hyphal length was estimated using the equation

$$R=\frac{\pi \,N\,A}{2\,H}$$

where *R* is the total hyphal length, *N* is the number of times hyphae crossed the horizontal lines on the grid, *A* is the area of one slide well, and *H* is the total length of the horizontal grid lines. Fungal biomass was estimated assuming 2.3 × 10^-6^ *g* C m^-1^ of hyphae (Frey et al., 1999).

### DNA EXTRACTION, PCR, AND PYROSEQUENCING

DNA was extracted from each 0.25 *g* sub-sample of soil using MoBio PowerSoil DNA extraction kit (MO BIO Laboratories, Inc., Carlsbad, CA, USA) following the instructions provided by the manufacturer. Eluted DNA samples were stored at –80°C before processing. The 16S and 18S rRNA genes were amplified for each sample using primer sets of F515/R806 (Bates et al., 2010) and SSU817R/SSU1196 (Borneman and Hartin, 2000), respectively, which were modified for the 454 pyrosequencing platform (Rousk et al., 2010).

Polymerase chain reactions were performed using 25 μL assays; 12.5 μL of KAPA2G Fast Multiplex Mix (Kapa Biosystems, Woburn, MA, USA), 1.25 μL of BSA (10.0 ng μL^-1^), 1.25 μL of each primer (10.0 μM), 8.5 μL of PCR grade water and 1.0 μL of a genomic DNA template (1.0 ng μL^-1^). The PCR thermal profile was developed following the protocols provided by the manufacturer (Kapa Biosystems, Woburn, MA, USA), which included an initial denaturation and enzyme activation step of 95°C for 3 min, followed by 30 cycles of 95°C for 10 s, 50°C for 10 s and 72°C for 1 s. PCR products were evaluated for amplification and their lengths by agarose gel electrophoresis, and purified with the UltraClean®; PCR Clean-UP Kit (MO BIO Laboratories, Inc., Carlsbad, CA, USA). Purified PCR products were quantified using Quant-iTTM PicoGreen®; (Invitrogen, Molecular Probes, Inc., Eugene, OR, USA) and pooled with an equal quantity of each PCR product for 16S and 18S separately following instructions by Selah Genomics (Greenville, SC, USA) where the pooled PCR products were sequenced on a Roche 454 FLX sequencer.

### SEQUENCING DATA PROCESSING

Sequences were processed using the QIIME 1.8.0 toolkit (Caporaso et al., 2010a). Sequences were assigned to operational taxonomic units (OTUs) at the ≥97% similarity level (Stackebrandt and Goebel, 1994). Taxonomy was assigned to each OTU via the Ribosomal Database Project (RDP, Wang et al., 2007; Cole et al., 2009) classifier for bacteria and NCBI BLAST (Johnson et al., 2008) for fungi. After singletons were removed, the remaining sequences were aligned using PyNAST (Caporaso et al., 2010b) and filtered to construct a phylogenetic tree using FastTree (Price et al., 2009). The bacterial and fungal sequences were normalized via random sub-sampling at 509 and 1136 reads per sample, respectively, for downstream analyses. We used UniFrac (Hamady et al., 2010), distant-based redundancy analysis (dbRDA, Legendre and Anderson, 1999), Phylocom (Webb et al., 2008), and four additional indices to assess differences in bacterial and fungal community composition. The four additional indices to assess microbial diversity included Shannon (Ludwig and Reynolds, 1988), observed OTUs, Chao 1 (Chao, 1984), and phylogenetic diversity (PD, Faith and Baker, 2007). UniFrac distances, both weighted (quantitative with relative abundances considered) and unweighted (qualitative with only presence or absence of OTUs considered), were computed among the samples and principal coordinate analyses (PCoA) were conducted using the QIIME. Using Phylocom analysis in the QIIME, we calculated net relatedness index (NRI) and nearest taxon index (NTI), which measure phylogenetic dispersion (Swenson, 2009). Relative bacterial abundance at the phylum and class levels and fungal abundance at the class level were used for dbRDA to assess relative contributions of taxa to the differences among the fertilization treatments, sites and soil horizons. Sequences were deposited to the MG-RAST server (metagenomics.anl.gov/) and are available to the public (accession numbers from 4574203.3 to 4574311.3, total 109 data sets).

### STATISTICAL ANALYSES

All computations were carried out using the *vegan* and *lme4* packages in R Development Core Team (2013). The *vegan* package was used for dbRDA. For the other statistical analysis, we used a mixed-effect analysis of variance (ANOVA) via the *lmer* function in the *lme4* package with the sites (i.e., the 1989 and 2006 sites), fertilization levels (i.e., control and high) and horizons (i.e., organic and mineral soils) as fixed effects, and blocks as a random effect. In all the mixed-effect ANOVA, we used average values of sub-samples nested within each plot, which was the experimental and statistical unit. A significance level of *P* ≤ 0.10 was employed to assess statistical significance due to high soil heterogeneity and relatively small sample sizes in this study, and all *P*-values are for two-sided confidence intervals.

## RESULTS

### MICROBIAL BIOMASS

**FIGURE 1:**
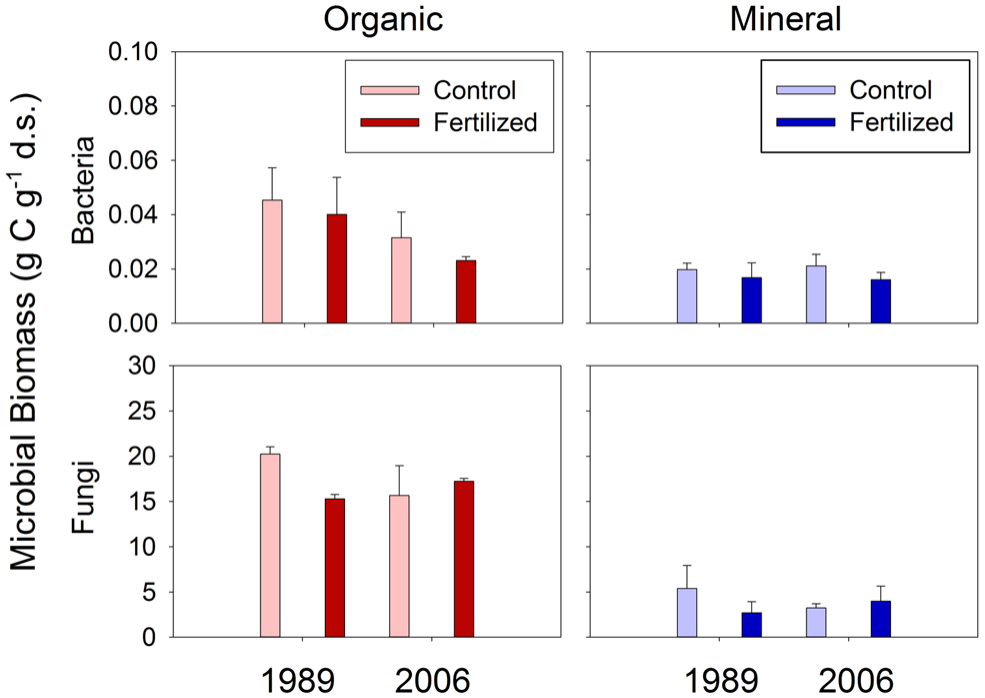
**Bacterial and fungal biomass in organic and mineral soil horizons.** There was no significant effect of treatment or site found by mixed-effect ANOVA.

Neither bacterial nor fungal biomass was affected by the fertilization treatments (**Figure 1**). Both bacterial and fungal biomass was greater in organic than mineral soils (**Figure 1**). Organic soils had 2.3 and 1.5 times greater bacterial biomass than mineral soils at the 1989 and 2006 sites, respectively (**Figure 1**). Organic soils had 4.4 and 4.5 times greater fungal biomass than mineral soils at the 1989 and 2006 sites, respectively (**Figure 1**).

### MICROBIAL COMMUNITY COMPOSITION

#### Bacterial community composition

**FIGURE 2:**
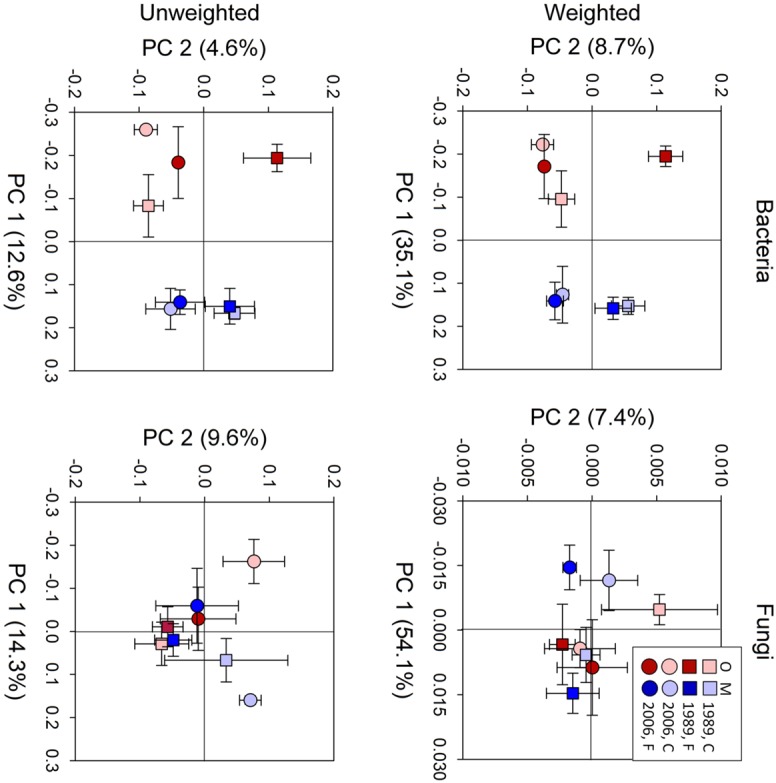
**Results of principle coordinate analysis of bacterial and fungal communities based on weighted and unweighted UniFrac distance metrics.** Statistical results using mixed-effect ANOVA to test effects of fertilization treatments, sites, soil horizons, and their interactions are shown in **Table 2**. Legend: O; organic soils, M; mineral soils, C; control, and F; fertilized.

**FIGURE 3:**
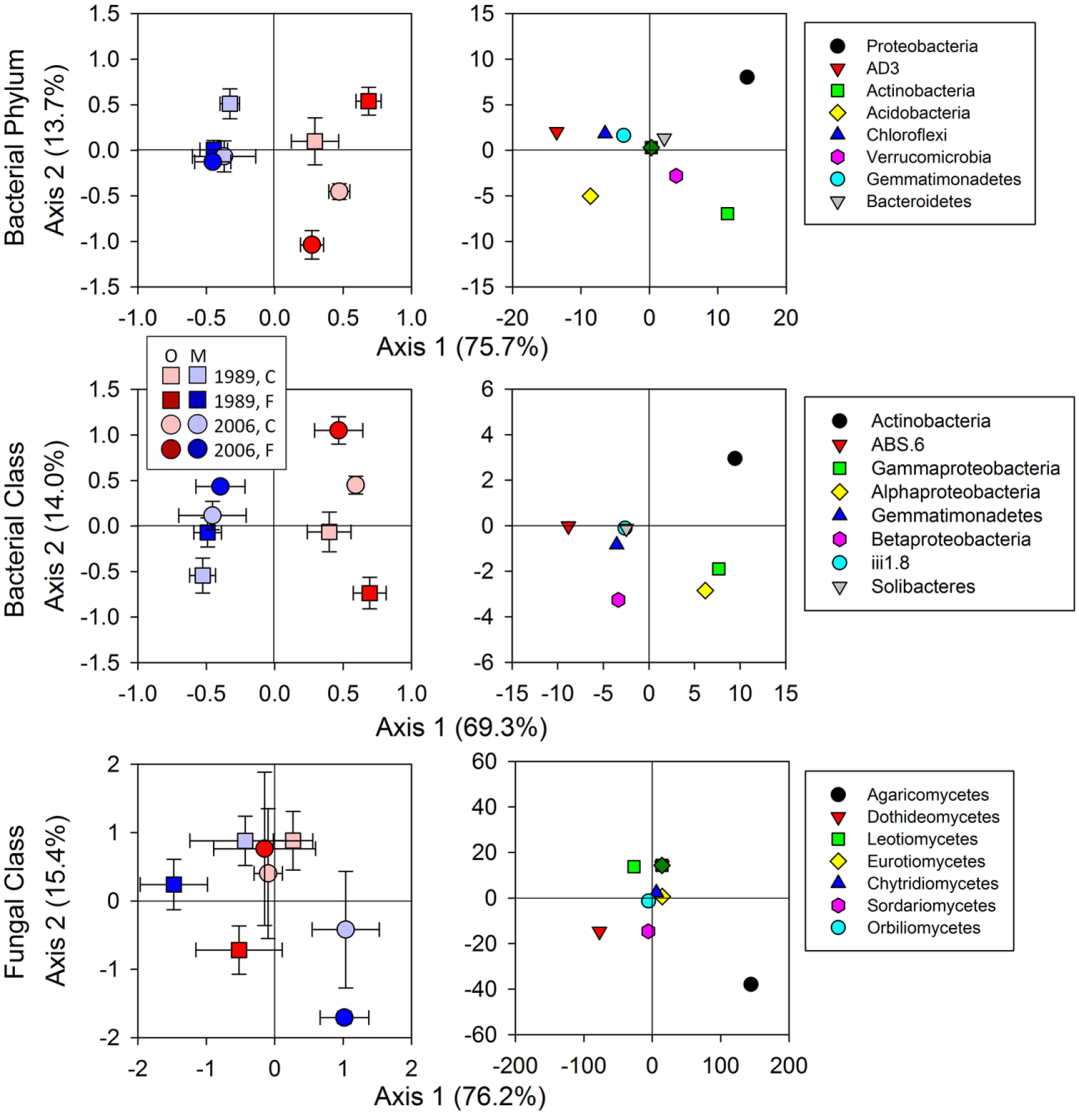
**Results of dbRDA for relative abundances in bacterial and fungal communities.** Bacterial communities were assessed at the Phylum and Class levels and fungal at the Class level only. Statistical results using mixed-effect ANOVA to test effects of fertilization treatments, sites, soil horizons, and their interactions are shown in **Table 3**. Legend: O; organic soils, M; mineral soils, C; control, and F; fertilized.

**FIGURE 4:**
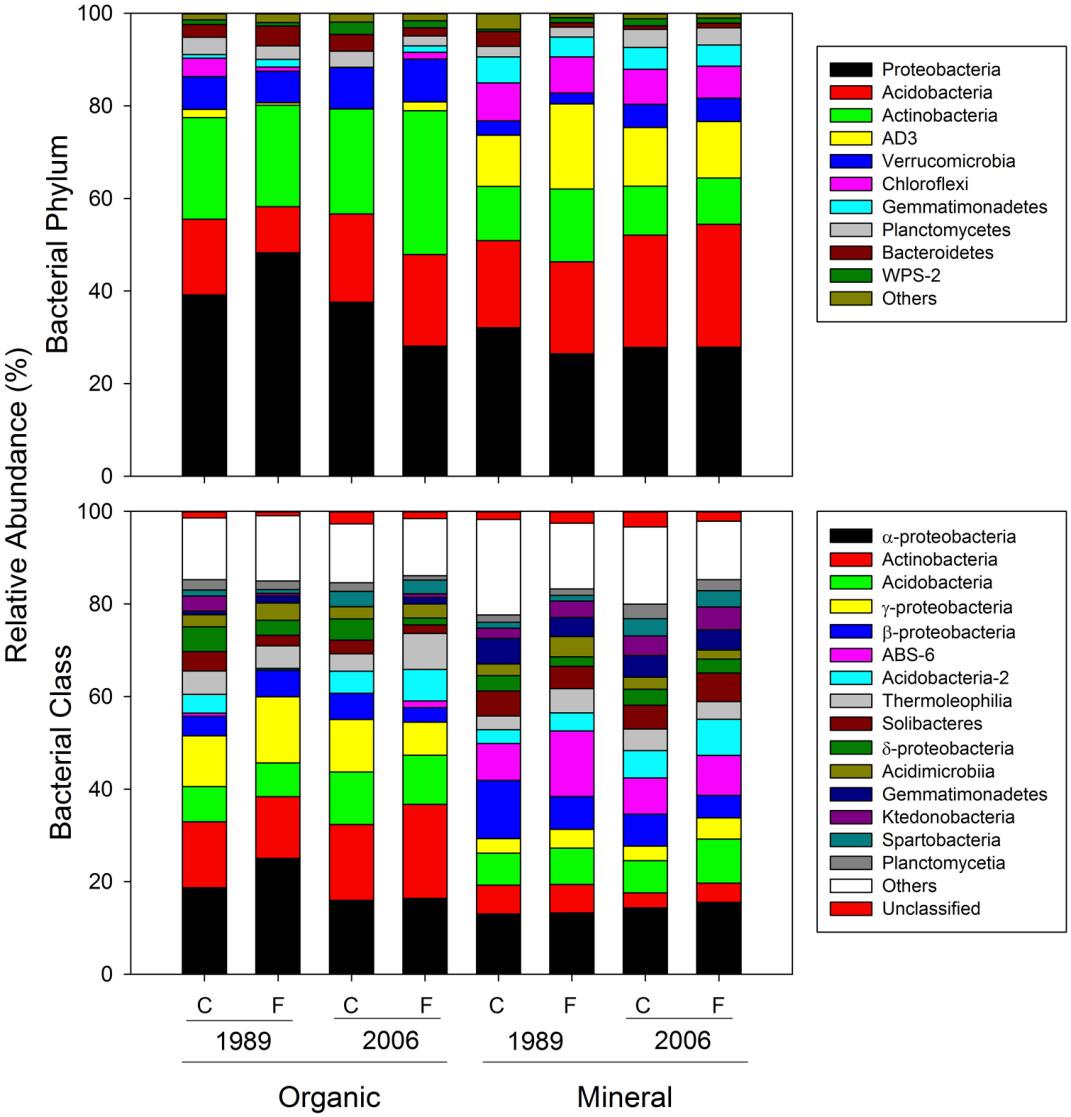
**Mean relative abundances of bacterial taxa at the phylum and class level**.

**FIGURE 5:**
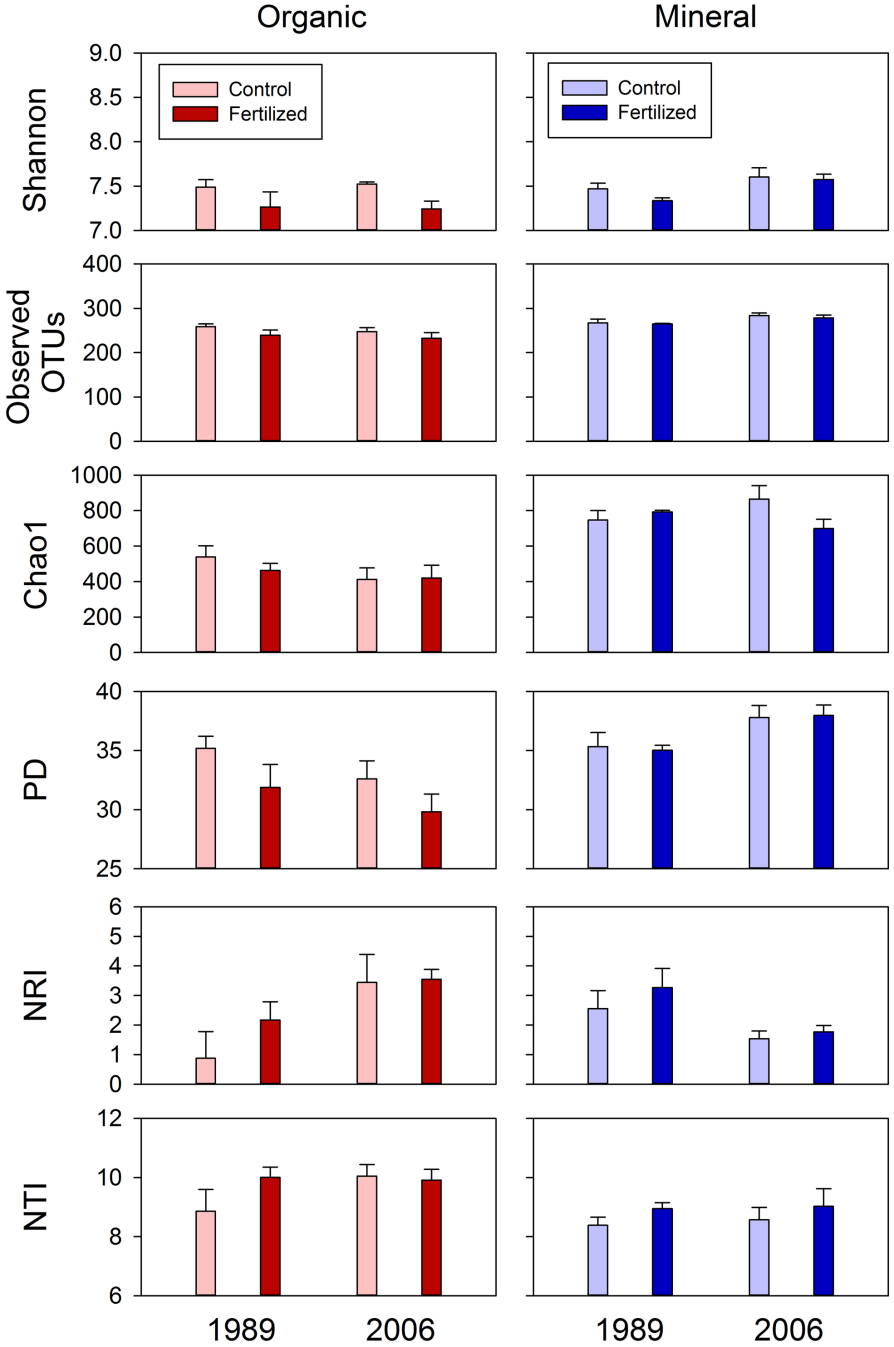
**Diversity (Shannon Index, observed OTUs and Chao 1 and PD) and dispersion (NRI and NTI) indices of bacterial communities for organic and mineral soils from control and fertilized treatments collected from the 1989 and 2006 sites.** Statistical results using mixed-effect ANOVA to test effects of fertilization treatments, sites, soil horizons, and their interactions are shown in **Table 4**.

Bacterial community composition at the OTU level was altered by fertilization in organic soils, but not in mineral soils (**Figure 2**). This alteration was supported by significant fertilization × horizon interaction effects in PC2 scores in both weighted and unweighted UniFrac (*P* = 0.006 and 0.023, respectively; **Table 2**) in combination with significant main fertilization effects in PC2 scores of weighted and unweighted UniFrac (*P* = 0.057 and 0.017, respectively; **Table 2**). The bacterial community alteration in organic soils was more pronounced in the 1989 site with a longer fertilization history than the 2006 site (**Figure 2**). This was supported by a significant three-way interaction (i.e., fertilization × site × horizon) for PC2 scores of weighted UniFrac (*P* = 0.017; **Table 2**). At the coarser phylogenetic levels of phylum and class, fertilization also altered bacterial community composition in organic soils, but not in mineral soils (**Figure 3**). However, the alteration in organic soils was not directional between the two sites. Fertilization increased Proteobacteria, and reduced Actinobacteria and Acidobacteria at the phylum level, and increased α-, β-, and γ-Proteobacteria and decreased Actinobacteria at the class level in relative abundances at the 1989 site, but those changes in relative abundance were opposite at the 2006 site (**Figures 3 and 4**). Those bi-directional alterations by fertilization in organic soils were supported by significant three-way interactions (i.e., fertilization × site × horizon) in axis 2 scores of dbRDA at the phylum (*P* = 0.016) and class (*P* = 0.020) levels (**Table 3**). Shannon Index was reduced by the fertilization treatments (*P* = 0.077; **Table 4**; **Figure 5**).

**Table 2:** Results of statistical analyses (*P*-values) for two primary PCoA scores from weighted and unweighted UniFrac for bacteria and fungi.

Independent variables	Weighted		Unweighted	
	PC1	PC2	PC1	PC2
**Bacteria**
F	0.935	**0.057**	0.789	**0.017**
S	0.405	**0.002**	0.344	**0.046**
H	**<0.001**	0.278	**<0.001**	0.285
F × S	0.171	**0.036**	0.177	0.245
F × H	0.778	**0.006**	0.823	**0.023**
S × H	0.362	0.676	0.131	0.914
F × S × H	0.112	**0.017**	**0.071**	0.119
**Fungi**
F	0.357	0.158	0.265	0.136
S	0.115	0.815	0.346	0.105
H	0.386	0.493	**0.026**	0.522
F × S	0.433	0.360	0.995	0.544
F × H	0.739	0.695	**0.026**	0.580
S × H	**0.005**	0.405	0.137	0.466
F × S × H	0.679	0.121	**0.031**	0.548

*F, S, and H represent fertilization, site and soil horizon, respectively. F × S, F × H, S × H, and F × S × H represent their corresponding interactions. *P*-values equal to or less than 0.100 are shown bold.*

**Table 3:** Results of statistical analyses (*P*-values) for two primary scores from dbRDA for bacteria and fungi.

Independent variables	Bacterial phylum		Bacterial class		Fungal class	
	Axis 1	Axis 2	Axis 1	Axis 2	Axis 1	Axis 2
F	0.768	0.218	0.806	0.224	0.290	0.138
S	0.759	**0.002**	0.681	**0.001**	**0.057**	0.267
H	**< 0.001**	**0.042**	**< 0.001**	0.172	0.696	0.166
F × S	0.135	0.301	0.275	**0.083**	0.325	0.497
F × H	0.364	0.421	0.814	0.127	0.893	0.669
S × H	0.800	**0.018**	0.875	**0.053**	**0.034**	**0.022**
F × S × H	**0.035**	**0.016**	0.083	**0.020**	0.863	0.126

*F, S, and H represent fertilization, site and soil horizon, respectively. F × S, F × H, S × H, and F × S × H represent their corresponding interactions. P-values equal to or less than 0.100 are shown bold.*

**Table 4:** Results of statistical analyses (*P*-values) for the indices to assess bacterial diversity.

Independent variables	Shannon	Observed OTUs	Chao 1	PD	NRI	NTI
**Bacteria**
F	**0.077**	0.136	0.267	0.164	0.204	0.195
S	0.250	0.616	0.437	0.856	0.606	0.363
H	**0.089**	**0.001**	**< 0.001**	**0.001**	0.611	**0.017**
F × S	0.869	0.947	0.428	0.799	0.367	0.353
F × H	0.188	0.292	0.740	0.125	0.765	0.991
S × H	0.172	**0.073**	0.226	**0.018**	**0.003**	0.556
F × S × H	0.521	0.781	**0.077**	0.989	0.692	0.406
**Fungi**
F	0.983	0.209	0.103	0.564	0.110	**0.048**
S	0.904	0.385	0.558	0.974	0.305	0.468
H	0.624	0.908	0.516	0.107	0.147	0.317
F × S	**0.071**	**0.015**	**0.021**	0.165	**0.019**	**0.038**
F × H	0.466	0.269	0.309	0.457	**0.068**	**0.031**
S × H	0.791	0.481	0.278	0.228	0.696	0.329
F × S × H	**0.049**	**0.031**	**0.085**	0.576	**0.031**	**0.021**

*F, S, and H represent fertilization, site and soil horizon, respectively. F × S, F × H, S × H, and F × S × H represent their corresponding interactions. *P*-values equal to or less than 0.100 are shown bold.*

Most of the variation in bacterial community composition at the OTU level was attributed to soil horizon (**Figure 2**; **Table 2**). There were significant differences due to soil horizon in PC1 scores of both weighted and unweighted UniFrac analyses (*P* < 0.001; **Table 2**), which explained 35.1 and 12.6% of variability, respectively, in the bacterial community composition at the OTU level (**Figure 2**). These differences in community composition between the two soil depths were also evident in relative abundances among taxa at the phylum and class levels assessed via dbRDA; main effects of the soil horizons were significant for axis 1 scores at both phylum and class levels (*P* < 0.001; **Table 3**), which explained 75.7 and 69.3% of variation, respectively (**Figure 3**). At the phylum level, the difference between the two soil horizons was primarily derived by relatively higher abundances of Proteobacteria (35.3 vs. 28.3% in organic and mineral soils, respectively) and Actinobacteria (27.7 vs. 11.1% in organic and mineral soils, respectively) in the organic soils and AD3 (0.9 vs. 13.0% in organic and mineral soils, respectively) and Acidobacteria (17.7 vs. 23.4% in organic and mineral soils, respectively) in the mineral soils (**Figures 3 and 4**). At the class level, the differences between soil horizons resulted from relatively higher abundances of Actinobacteria (15.8 vs. 5.3% in organic and mineral soils, respectively), γ-Proteobacteria (11.5 vs. 3.8% in organic and mineral soils, respectively) and α-Proteobacteria (20.2 vs. 14.2% in organic and mineral soils, respectively) in the organic soils and ABS 6 (0.5 vs. 10.0% in organic and mineral soils, respectively) in the mineral soils (**Figures 3 and 4**). The two soil horizons were also significantly different in diversity evident in observed OTUs (*P* < 0.001), Chao 1 (*P* < 0.001) and PD (*P* = 0.001) and phylogenetic dispersion measured as NTI (*P* = 0.017; **Figure 5**; **Table 4**). Observed OTU’s and scores of Chao 1 and PD were significantly lower for the organic than mineral soils, and NTI scores were higher for the organic than mineral soils (**Figure 5**; **Table 4**).

The 1989 and 2006 sites had inherently different bacterial community compositions, indicated by significant main site effects in PC2 scores of weighted and unweighted UniFrac (*P* = 0.002 and 0.046, respectively; **Table 2**) as well as axis 2 scores of dbRDA at both phylum and class levels (*P* = 0.002 and 0.001, respectively; **Table 3**). At the phylum level, the 1989 site had a relatively greater abundance of Proteobacteria and lower abundance of Actinobacteria than the 2006 site (**Figures 3 and 4**). At the class level, the 1989 site had a relatively higher abundance of α- and γ-Proteobacteria, and a lower abundance of Actinobacteria than the 2006 site (**Figures 3 and 5**).

Almost all of the bacterial 16S rRNA amplicons were identified at least to the phylum level (30027/30031; **Figure 4**) and 98.0% to the class level (29444/30031; **Figure 4**). At the phylum level, Proteobacteria was the most dominant taxon comprising 33.1%, followed by Acidobacteria (19.3%), Actinobacteria (18.2%), and AD3 (6.9%, **Figure 4**). These four phyla constituted 78.7% of the overall abundance of bacteria found in this study (**Figure 4**). In organic soils, Proteobacteria (35.3%), Acidobacteria (17.7%), and Actinobacteria (27.7%) constituted 80.7% (**Figure 4**). In mineral soils, Proteobacteria (28.3%), Acidobacteria (23.4%), AD3 (13.0%) and Actinobacteria (11.1%) constituted 75.8% (**Figure 4**). At the class level, α-Proteobacteria was most abundant across the soil horizons, constituting 17.2% in the overall abundance (**Figure 4**). In the organic soils, α-Proteobacteria (20.2%), Actinobacteria (15.8%) and γ-Proteobacteria (11.5%) were three most abundant taxa, constituting 47.5% of the overall abundance in the soil horizon (**Figure 4**). In the mineral soils, α-Proteobacteria (14.2%), ABS-6 (10.0%) and β-Proteobacteria (8.4%) are three most abundant taxa, constituting 32.7% of the overall abundance of bacteria found in the mineral soils (**Figure 4**).

#### Fungal community composition

**FIGURE 6:**
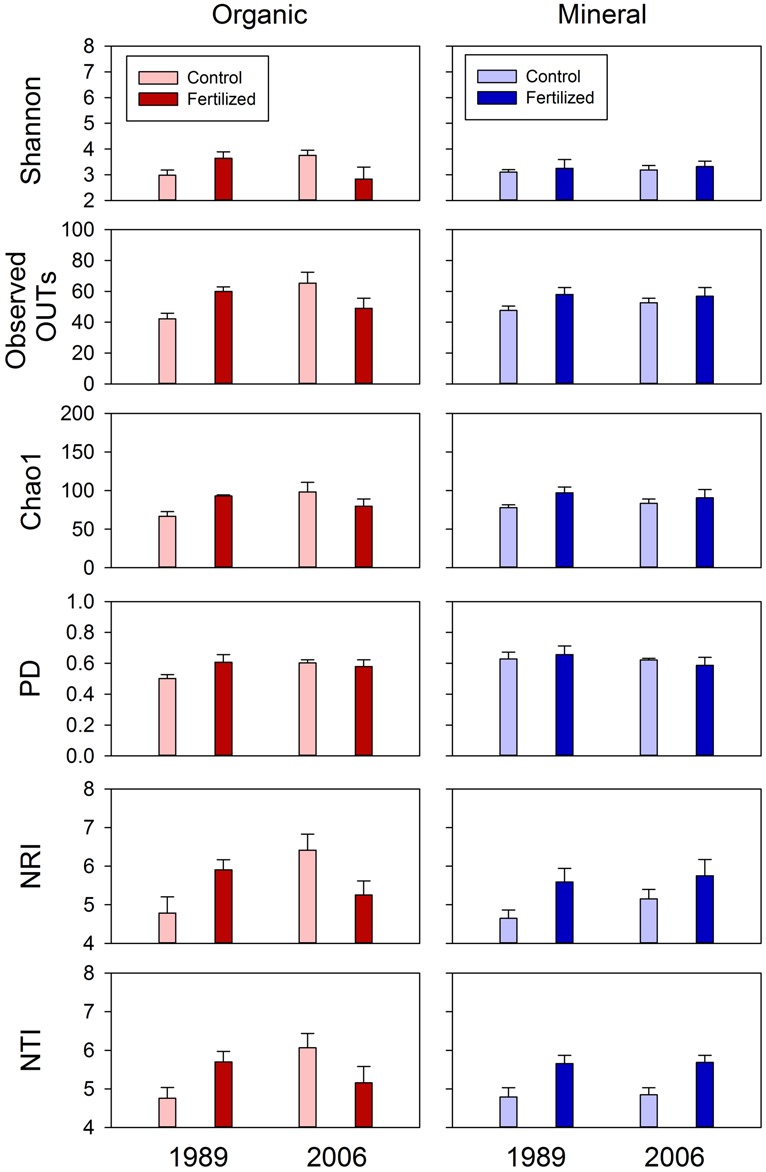
**Diversity (Shannon Index, observed OTUs and Chao 1 and PD) and dispersion (NRI and NTI) indices of fungal communities for organic and mineral soils from control and fertilized treatments collected from the 1989 and 2006 sites.** Statistical results using mixed-effect ANOVA to test effects of fertilization treatments, sites, soil horizons, and their interactions are shown in **Table 4**.

The fertilization treatments did not influence fungal community composition. None of the primary scores from UniFrac or dbRDA had a significant main effect of fertilization (**Tables 2 and 3**). No significant fertilization effect was found for any of the diversity or phylogenetic dispersal indices, except NTI which was increased by fertilization (*P* = 0.048; **Table 4**; **Figure 6**).

The soil horizons were not a strong driver of fungal community composition structure as the only significant horizon effect was found in PC1 scores of unweighted UniFrac (P = 0.026; **Table 2**; **Figure 2**). There was no significant main effect of the soil horizons for the diversity or dispersal indices (**Figure 6**; **Table 4**).

Three diversity indices (Shannon Index, observed OTUs and Chao 1) and the two dispersion indices (NRI and NTI) showed similar trends in fungal community composition (**Figure 6**). Fertilization consistently increased these indices in mineral soils in both 1989 and 2006 sites (**Figure 6**). However, such consistent fertilization effects were not observed in organic soils; fertilization increased these indices in the 1989 site, but decreased them in the 2006 site (**Figure 6**). These site- and horizon-dependent fertilization effects were supported by significant fertilization × site interaction effects for Shannon Index (*P* = 0.071), observed OTUs (*P* = 0.015), Chao 1(*P* = 0.021), NRI (*P* = 0.019) and NTI (*P* = 0.038), significant fertilization × horizon interaction effects for NRI (*P* = 0.068) and NTI (*P* = 0.031), and significant three-way interaction effects (i.e., fertilization × site × horizon) for Shannon Index (*P* = 0.049), observed OTUs (*P* = 0.031), Chao 1 (*P* = 0.085), NRI (*P* = 0.031), and NTI (*P* = 0.021; **Table 4**).

**FIGURE 7:**
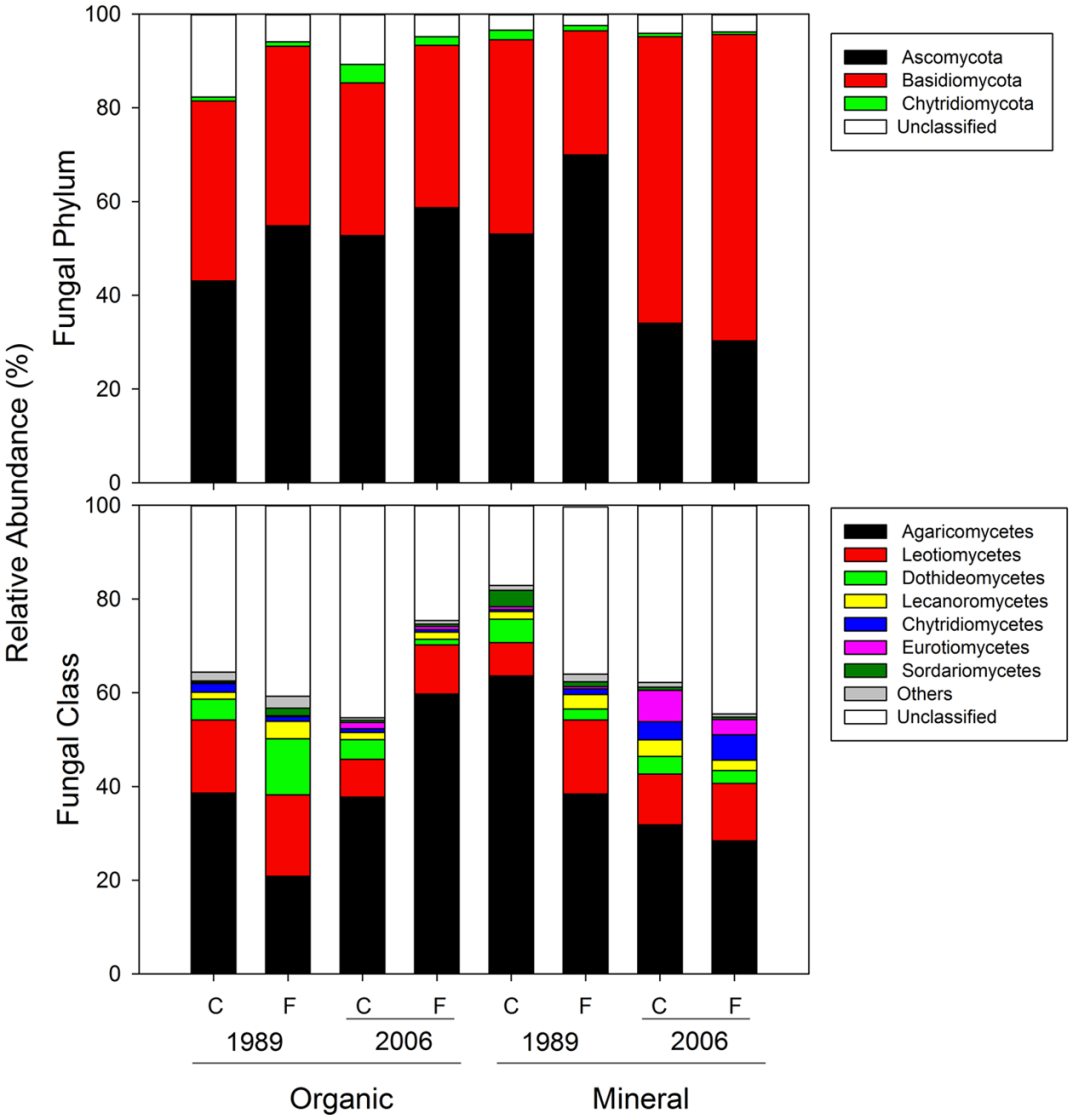
**Mean relative abundances of fungal taxa at the phylum and class levels for organic and mineral soils from control and fertilized treatments collected from the 1989 and 2006 sites**.

For fungal 18S rRNA amplicons, 93.0% could be identified at least at the phylum level (66587/71568; **Figure 7**) and 63.8% at the class level (45673/71568; **Figure 7**). At the phylum level, Ascomycota and Basidiomycota constituted 50.5 and 40.7% of overall abundances, respectively (**Figure 7**). At the class level, Agaricomycetes was most abundant (38.7%) followed by Leotiomycetes (11.5%) and Dothideomycetes (5.5%; **Figure 7**).

#### Relative abundances of ectomycorrhizal taxa

**FIGURE 8:**
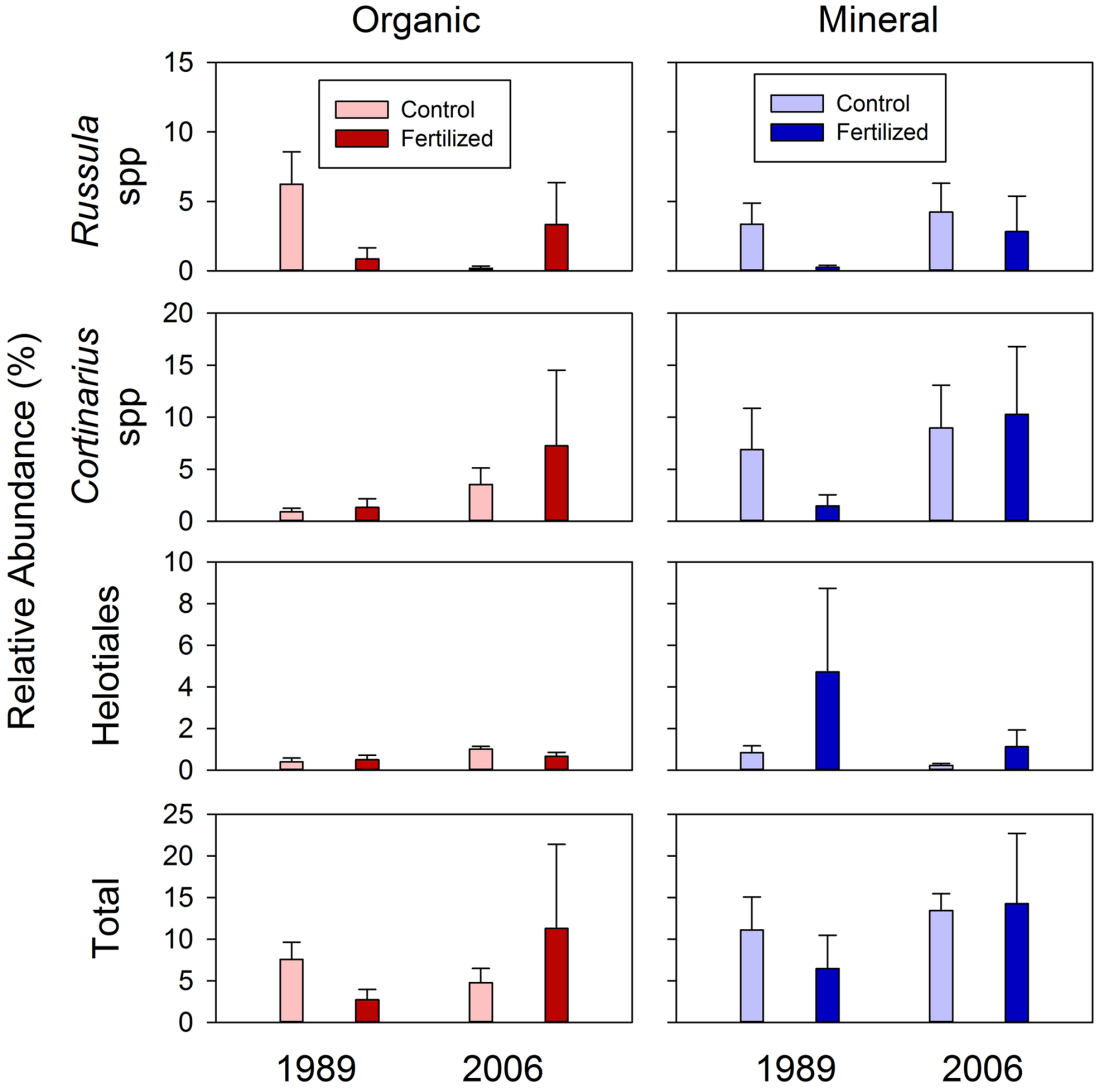
**Relative abundances of ectomycorrhizal taxa, *Russula* and *Cortinarius* spp., and members of the order *Helotiales* among rarefied fungal taxa for organic and mineral soils from control and high fertilized treatments collected from the 1989 and 2006 sites.** Statistical results using mixed-effect ANOVA to test effects of fertilization treatments, sites, soil horizons and their interactions are shown in **Table 5**.

The relative abundances of mycorrhizal fungi among all fungal OTU’s are shown in **Figure 8**. Three taxa of mycorrhizal fungi were detected among the sequenced fungal taxa; two ectomycorrhizal taxa of *Russula* and *Cortinarius* spp. (Newsham et al., 2009), and members of the order *Helotiales* which include many ericoid mycorrhizal spp. (Walker et al., 2011). There was a significant effect of the soil horizons for the abundances of *Cortinarius* spp. and the total ectomycorrhizal taxa (*P* = 0.076 and 0.061, respectively; **Table 5**). This suggested that relative abundances of *Cortinarius* spp. and the three ectomycorrhizal taxa combined were higher in mineral than organic soils among all fungal OTU’s found in this study (**Figure 8**).

**Table 5:** Results of statistical analyses (*P*-values) for the indices to assess relative abundances of ectomycorrhizal taxa.

Independent variables	*Russua* spp.	*Cortinarius* spp.	*Helotiales*	Total
F	0.163	0.411	0.338	0.169
S	0.966	0.357	0.706	0.527
H	0.566	**0.076**	0.447	**0.061**
F × S	0.112	0.788	0.652	0.273
F × H	0.319	0.548	0.192	0.942
S × H	0.154	0.602	0.118	0.364
F × S × H	0.212	0.366	0.878	0.634

*F, S, and H represent fertilization, site and soil horizon, respectively. F × S, F × H, S × H, and F × S × H represent their corresponding interactions. *P*-values equal to or less than 0.100 are shown bold.*

## DISCUSSION

Long-term fertilization altered bacterial community composition to a greater degree in the organic than mineral soils, and at the 1989 compared to the 2006 site. The change in bacterial composition was accompanied by a reduction in evenness among OTUs. Long-term fertilization at the 1989 site increased copiotrophic α- and β-Proteobacteria, and decreased oligotrophic Acidobacteria as predicted, most likely as a result of increased labile C input via rhizodeposition caused by stimulated net primary productivity of vascular plants (Ramirez et al., 2010). These bacterial responses to fertilization were consistent with our *a priori* predictions based on the generalized ecology of these taxa with respect to nutrient and C availability (Fierer et al., 2007). In contrast to the bacteria community, fungal community composition was not as sensitive to the fertilization treatments. The insensitivity of the fungal communities included the mycorrhizal taxa associated with shrubs, which became dominant in response to fertilization. This fungal response to fertilization is different from results found in warming experiments using greenhouses in the same ecosystem (e.g., Deslippe et al., 2012). Though fertilization is often used as a means to mimic an effect of warming (i.e., increased nutrient availability in soils, Mack et al., 2004; Aerts, 2010), the fungal dynamics in response to fertilization do not appear to emulate the fungal dynamics under warming in Arctic tundra (Rinnan et al., 2007, 2013).

### BACTERIAL COMMUNITIES

Reduction in the Shannon Index for bacterial communities by the fertilization treatments was significant (**Figure 6**; **Table 4**). Given no significant difference in observed numbers of OTUs between the treatments (**Figure 6**; **Table 4**), the fertilization treatments reduced evenness of bacterial community compositions at the OTU level (Ludwig and Reynolds, 1988). These observations are consistent with long-term fertilization experiments in several ecosystems, including Arctic tundra (24-year fertilization, Campbell et al., 2010), grassland (20-year fertilization, Coolon et al., 2013), coniferous forest (10-year fertilization, Burke et al., 2006), and agricultural fields including pasture (12-year fertilization, Jangid et al., 2008) and corn fields (*Zea mays*; 8-year fertilization, Ramirez et al., 2010). However, reduction of microbial diversity by fertilization is not a universal phenomenon; a fertilization experiment in a hardwood forest at Harvard Forest in Massachusetts, USA, showed increased bacterial diversities (20-year fertilization, Turlapati et al., 2013), and no significant change was found in a grassland ecosystem at Cedar Creek in Minnesota, USA (27-year fertilization, Ramirez et al., 2010; Fierer et al., 2012).

Bacterial community composition at the phylum level was significantly different between the organic and mineral soil horizons (**Figure 3**; **Table 3**), as organic soils had a relatively higher abundance of Bacteroidetes and a lower abundances of Acidobacteria (**Figures 3 and 4**). The differences in the relative abundances can be explained by differences in the availability of C substrates within the two soil horizons (Fierer et al., 2007): the organic soils had more organic C than the mineral soils (**Table 1**), thus copiotrophic Bacteroidetes and oligotrophic Acidobacteria were expected to be relatively more abundant in the organic and mineral soils, respectively. This was also the case for copiotrophic α-Proteobacteria, which were relatively more abundant in the organic soils compared to the than mineral soils (**Figures 3 and 4**). However, β-Proteobacteria were more abundant in the mineral than organic soils even though the phylum is considered copiotrophic (**Figures 3 and 4**; Fierer et al., 2007). These differences in the bacterial community composition between the organic and mineral soils in this study are consistent with those reported by Campbell et al. (2010) who investigated bacterial community structures using high-throughput sequencing in the same study area. However, in contrast to the results found at both 1989 and 2006 sites in this study (**Figure 3**) and by Wallenstein et al. (2007), Campbell et al. (2010) found that bacterial community composition was relatively similar between shrub organic and mineral soils at the phylum/class levels at this study area.

The responses of bacterial phylum and class community composition to fertilization were not uniform across sites, fertilization histories or soil horizons, as indicated by the a significant three-way interaction effect of fertilization × site × horizon in axis 2 scores of dbRDA for both bacterial phylum and class (**Table 3**; **Figure 3**). The organic soils from the fertilized plots within the1989 site, which were expected to be most affected by fertilization because of their position and longer treatment history, had a lower relative abundance of oligotrophic Acidobacteria, and higher relative abundances of copiotrophic α-, β-, and γ-Proteobacteria than soils from the control plots within the site (**Figures 3 and 4**). These observations supported our first prediction that fertilization should increase the relative abundance of copiotrophs and reduce the abundance of oligotrophs (Cleveland et al., 2007; Fierer et al., 2007, 2012; Nemergut et al., 2010). We cannot separate a direct effect of increased N and P from an indirect effect via plants (i.e., increased productivity and community shift) on the altered bacterial community compositions. However, we can speculate that the consistent shifts in community compositions of the copiotrophs and oligotroph were likely caused by increased net primary productivity of vascular plants (Fierer et al., 2007; Ramirez et al., 2010), especially shrubs (Chapin and Shaver, 1985; Chapin et al., 1995; Shaver et al., 2001). We ruled out a potential mechanism that fertilization stimulated microbial production of C-degrading extracellular enzymes (Koyama et al., 2013), resulting in increased C availability, which in turn altered bacterial community composition. Koyama et al. (2013) found that fertilization consistently increased C-degrading extracellular enzyme activities in organic horizons of both 1989 and 2006 sites, but bacterial community shifts at the phylum and class levels were opposite between the two sites (**Figure 3**). Another mechanism that fertilization increased below-ground litter input was also unlikely as Sullivan et al. (2007) demonstrated that long-term fertilization decreased root production at the community level. Our finding of bacterial community shift with fertilization in the 1989 site was consistent with Campbell et al. (2010) who found increased relative abundances of α- and γ-Proteobacteria and a reduced relative abundance of Acidobacteria by a 24-year fertilization in the same ecosystem.

The results from analyses using finer taxonomic levels, including UniFrac, need to be interpreted with caution, given seasonal variation in communities and the single sample date used in this study. Even though bacterial community compositions are relatively stable across seasons in this ecosystem (e.g., Deslippe et al., 2012) especially at coarse taxonomic levels (e.g., phylum), they can be different in finer taxonomic levels (Wallenstein et al., 2007).

### FUNGAL COMMUNITIES

We found that fertilization did not significantly affect the relative abundance of mycorrhizal fungi detected in this study (**Figure 8**; **Table 5**). These findings contradicted our prediction that the fertilization treatments had increased relative abundances of these ectomycorrhizal fungi associated with shrubs, including *B. nana*, which became dominant in response to fertilization. There are three possible explanations for this observation; (1) fertilization reduced infection rates of ectomycorrhizal fungi in shrub roots; (2) increased nutrient availability did not increase below-ground shrub biomass; and (3) fertilization increased saprotrophic fungi, which in turn reduced relative abundances of ectomycorrhizal fungi. The first explanation was consistent with a finding by Urcelay et al. (2003) who reported that ectomycorrhizal infection rates of *B. nana* root were reduced almost by half following 3-year fertilization in this study area. Both *Russula* and *Cortinarius* spp. are considered nitrophobic and have been shown to decrease in abundance when N availability is high (Lilleskov et al., 2002). Other studies have shown that mycorrhizal infection rates often decrease following fertilization in many ecosystems. In a meta-analysis, Treseder (2004) found that fertilization of N and P tended to decrease mycorrhizal abundances across biomes, though this meta-analysis did not include Arctic tundra. The second explanation was supported by Gough et al. (2012) who reported that an 11-year fertilization treatment significantly increased above-ground plant biomass, especially *B. nana*, without significant change in below-ground plant biomass in moist acidic tundra adjacent to our study sites. Such lack of change in below-ground biomass was most likely caused by reduced allocation of resources to roots by plants with abundant nutrients (Treseder and Vitousek, 2001). We ruled out the third explanation given that the fungal biomass between the two treatments did not differ significantly (**Figure 1**). Taken together, our finding of no change in relative abundances of ectomycorrhizal fungi was most likely caused by little change in root biomass of shrubs and reduced infection rates in shrub roots under increased nutrient availability.

### COMPARISON WITH WARMING EFFECTS ON ARCTIC TUNDRA ECOSYSTEMS

Fertilization, typically N and P, has been used to obtain insights for ecosystem responses to warming in Arctic tundra ecosystems (Mack et al., 2004; Aerts, 2010); warming stimulates SOM decomposition, resulting in increased nutrient availability for plants and soil microbes (Chapin et al., 1995; Hartley et al., 1999; Rustad et al., 2001; Schmidt et al., 2002; Schimel et al., 2004; Aerts et al., 2006). In a few Arctic tundra ecosystems, warming and fertilization showed similar effects on above-ground vegetation dynamics (e.g., Michelsen et al., 1996) and ectomycorrhizal fungal abundance (Clemmensen et al., 2006). However as noted below, these cases appear to be exceptions rather than rules. Warming and fertilization experiments tended to show different effects on plant community composition (Chapin et al., 1995; Press et al., 1998; Campioli et al., 2012), above-ground biomass (Chapin et al., 1995; Sorensen et al., 2008), soil microbial biomass (Rinnan et al., 2013), soil microbial community composition (Deslippe et al., 2011), and carbon storage (Mack et al., 2004; Sistla et al., 2013). Thus, fertilization does not appear to be an appropriate proxy for warming in this ecosystem.

### MICROBIAL DIVERSITY AND STABILITY IN ECOSYSTEM PROCESSES

High plant community diversity can stabilize temporal productivity via species asynchrony (Tilman et al., 2006; Isbell et al., 2009; Hector et al., 2010); as environmental variability reduces productivity of some plant species, other species compensate the reduction. This type of response has been observed in Arctic tussock and wet meadow tundra in the same site as this study was conducted (Chapin and Shaver, 1985). Chapin and Shaver (1985) observed that community level above-ground net primary productivity was relatively stable from year to year, even though production of individual plant species showed great variation along with environmental fluctuations over time. Species asynchrony in these communities was supported by an environmental manipulation experiment that demonstrated no single factor limiting productivity of all the species in the same study site (Chapin and Shaver, 1985). Fertilization experiments that included a network of 41 different grassland sites demonstrated that increased nutrient availability decreased stability in temporal productivity, not because species diversity at the sites decreased, but because fertilization had increased temporal variability in productivity and, at the same time, decreased asynchrony of diverse plant communities, which would otherwise stabilize plant biomass production over time (Hautier et al., 2014).

How the concept of species asynchrony applies to soil microbial diversity and its effects on the stability of ecosystem processes mediated by soil microbes is unclear. In a meta-analysis, Allison and Martiny (2008) showed that microbial communities altered by disturbances often change ecosystem process rates, suggesting a potential relationship between microbial diversity and stability. In this study, fertilization reduced soil bacterial diversity, indicated by a reduced Shannon Index (**Figure 5**; **Table 4**). In particular, the relative abundance of oligotrophic Acidobacteria was reduced, and that of copiotrophic taxa of Proteobacteria was increased in the organic soils in the longer fertilization (**Figures 3 and 4**). These observed changes in microbial diversity could contribute to increased potential activities of C-degrading enzymes and altered stoichiometry between C- and N-degrading enzyme activities in the same soils used to assess microbial community structures in this study (Koyama et al., 2013). However, we do not know how these changes in microbial diversity and processes (e.g., extracellular enzyme production) result in short-term (seasonal within a year) and long-term (year to year) temporal stability of ecosystem processes. Two characteristics of soil microbes make it challenging to predict the relationship; (1) higher diversity of soil microbes than plant species and (2) microbial dormancy. One gram of soil can contain more than 10,000 OTUs of bacteria (Roesch et al., 2007) and their functions can be redundant (Allison and Martiny, 2008). Thus, extreme abundance and diversity, and functional redundancy of soil microbes can buffer change in stability in ecosystem processes caused by reduced microbial diversity. In addition, not all the microbes found by the method employed in this study were active at a given time. McMahon et al. (2011) demonstrated that active bacterial communities were different between summer and winter in shrub soils in the same study site. This microbial dormancy will add another complexity to assess microbial diversity and its effects on stability in ecosystem processes.

## CONCLUSION

Given the dominant role of N in limiting both plant and microbial activity in Arctic tundra, it was not surprising that long-term fertilization strongly affected bacterial diversity and community composition. Long-term fertilization reduced bacterial evenness at the OTU level, increased copiotrophic classes (α-Proteobacteria and β-Proteobacteria) and reduced a dominant oligotrophic phylum (Acidobacteria). This diverse soil bacterial community plays a critical role in cycling nutrients, including N, stored in SOM of varying recalcitrance in Arctic tundra. Thus, changes in the composition of this community due to N availability are likely to impact SOM cycling. On the other hand, chronic fertilization did not significantly affect fungal community composition, including ectomycorrhizal fungi, despite increases in the abundance of their host plants (e.g., *B. nana*). This insensitivity of ectomycorrhizal fungi suggests reduced resource allocation to below-ground of their host plants in response to the chronic fertilization. Nitrogen appears to affect bacteria and fungi in different ways, indicating that changes in N availability could restructure below-ground communities and food webs.

## Conflict of Interest Statement

The authors declare that the research was conducted in the absence of any commercial or financial relationships that could be construed as a potential conflict of interest.
